# Supplementary material for: Molecular Characterisation of Epstein–Barr Virus in Classical Hodgkin Lymphoma
Source: Int J Mol Sci. 2022 Dec 9;23(24):15635. doi: 10.3390/ijms232415635 (PMC9778902; doi:10.3390/ijms232415635)
Supplement: Supplementary file 1 [file ijms-23-15635-s001.zip › ijms-2034426-supplementary.pdf]

International Journal of *Molecular Sciences*

MDPI

Table S1. Available clinical data for 46 cHL patients included in this study.

Sample	Sex	Age	Histological Subtype	Clinical Stage	Therapy	Follow Up (Months)	Remission
cHL1	M	24	NS	II A	4x ABVD, radiation	67	complete
cHL2	M	58	MC	IV B	2 x escBEACOPP, 4x ABVD	99	complete
cHL3	F	79	MC	n. a.	n. a.	n. a.	n. a.
cHL5	M	18	MC	II A	6X ABVD	64	complete
cHL6	M	76	MC	III A	4X CHLVPP	88	complete
cHL7	F	73	NS	I A	6 x ABVD	85	complete
cHL9	M	43	ND	IV B	6x ABVD	86	complete
cHL10	M	43	ND	n. a.	n. a.	n. a.	n. a.
cHL11	M	31	NS	IV A	6xesBEACOPP	86	complete
cHL12	M	75	NS	n. a.	n. a.	n. a.	n. a.
cHL13	F	77	TR	II B	R Bendamustin, only 3x	5	n. a.
cHL14	M	54	MC	III B	6x esc BEACOPP	36	complete
cHL15	M	69	NS	I A	n. a.	4	n. a.
cHL16	M	77	NS	n. a.	n. a.	n. a.	n. a.
cHL17	F	84	NS	n. a.	n. a.	0	n. a.
cHL18	F	52	NS	II B	5 x ABVD	34	complete
cHL19	F	70	MC	IV A	LVPP	11	n. a.
cHL21	F	40	MC	III A	6 x ABVD	48	complete
cHL22	F	60	MC	III B	6x COPP	75	complete
cHL24	M	59	MC	III B	6 x ABVD	72	complete
cHL25	M	40	MC	I A	6 x ABVD	69	complete
cHL26	M	57	NS	I A	4 x ABVD	72	complete
cHL27	M	67	TR	n. a.	n. a.	n. a.	n. a.
cHL28	M	61	ND	n. a.	n. a.	n. a.	n. a.
cHL29	M	51	NS	IV B	4 xescBEACOPP	64	complete
cHL30	M	69	ND	II B	without therapy	7	/
cHL31	M	23	NS	n. a.	n. a.	n. a.	n. a.
cHL32	M	60	MC	n. a.	n. a.	n. a.	n. a.
cHL33	M	64	LD	IV B	6x ABVD + mediastinum radiation	55	complete
cHL34	M	25	LR	n. a.	6 x ABVD	36	n. a.
cHL35	M	53	MC	III A	6x ABVD	55	complete
cHL36	M	56	MC	n. a.	n. a.	n. a.	n. a.
cHL38	M	65	MC	III B	2 x AVD	10	n. a.
cHL39	M	53	MC	IIA	6 x ABVD	50	complete
cHL40	M	59	ND	III A	6x ABVD, radiation	41	complete
cHL41	F	72	MC	n. a.	n. a.	n. a.	n. a.
cHL42	M	77	MC	n. a.	n. a.	n. a.	n. a.
cHL43	F	51	MC	II B	6X ABVD	41	complete
cHL44	M	56	LR	II A	4x ABVD, radiation	37	complete
cHL45	F	81	TR	n. a.	n. a.	n. a.	n. a.
cHL46	M	10	ND	n. a.	n. a.	n. a.	n. a.
cHL47	F	39	ND	III A	4x esc BEACOPP	36	complete
cHL48	M	20	ND	IV B	1x ABVD, 1x esc BEACOPP	7	/
cHL49	M	87	MC	n. a.	n. a.	n. a.	n. a.
cHL50	M	55	ND	IV A	2xABVD, 4xBEACOPP	29	complete
cHL52	M	52	LR	n. a.	2009. CH II A 3x ABVD radiation, 3x ICE ALLO transplantation	5	n. a.

LR - lymphocyte rich, LD - lymphocyte depleted, MC - mixed cellularity, NS - nodular sclerosis, TR - SLL transformations to cHL, ND – subtype not determined, ABVD - doxorubicin hydrochloride (adriamycin), bleomycin sulfate, vinblastine sulfate, and dacarbazine, escBEACOPP - escalated-

dose bleomycin, etoposide, doxorubicin, cyclophosphamide, vincristine, procarbazine, and prednisone, CHLVPP - chlorambucil, vinblastine, procarbazine and prednisolone, LVPP - lemustine, vinblastine, procarbazine and prednisone, COPP - cyclophosphamide, oncovin, procarbazine and prednison., ICE ALO transplantation – ifosfamide, carboplatin, etoposide and allotransplantation, R - rituximab, n. a. – not available.

Table S2. Primers used for *EBNA3C* and *LMP1* amplification.

Gene	Primers	Amplicon Lenght
EBNA3C [53]	F: 5′-AGA AGG GGA GCG TGT GTT GT-3′<br>R :5′-GGC TCG TTT TTG ACG TCG GC-3′	153/246 bp
LMP1 [13]	F: 5′ CGG AAG AGG TGG AAA ACA AA -3′<br>R: 5′- GTG GGG GTC GTC ATC ATC TC -3′	131/161 bp

Table S3. Distribution of EBV genotypes 1 and 2 in patients with EBV-associated diseases.

Study	Experimental Model	Study Design	Main Results	Conclusions
Kaymaz et al, 2020	- eBL in children (Kenya)	eBL: n=58<br>healthy controls: n=40	- significantly increased EBV type 1 prevalence in eBL patients (74.5%) compared with healthy children (47.5%) (p = 0.007)<br>- three novel intertypic recombinants carrying type 1 EBNA2 and type 2 EBNA3 regions and one new genome with a 20 kb deletion were detected	- complexity of the EBV population structure in eBL is emphasised
Van Baarle et al, 2000	- MSM (incl. HIV-1-positive) and heterosexual healthy men (Netherlands)	HIV-1-infected MSM: n=85<br>HIV-1-negative MSM: n=113<br>HIV-1-negative heterosexual men: n=114	- significantly higher prevalence of EBV type 2 among MSM compared with heterosexuals (39% vs. 6%) and in high versus low risk heterosexual men (15% vs. 0)<br>- EBV type 2 infection was independently associated with HIV-1 infection and number of sex partners (multivariate analyses)	- EBV type 2 infection is more prevalent among Caucasian MSM and is associated with sexual transmission
Crawford et al, 2006	- a prospective study, a cohort of university students, Edinburgh	EBV seronegative young adults: n=510, follow-up period of 3 years	- EBV type 1 was significantly overrepresented in IM, compared with students that seroconverted but did not develop IM (p = 0.001)<br>- penetrative sexual intercourse was a risk factor for EBV seroconversion (p = 0.004)	- EBV type 1 infection is more likely to result in IM
Higgins et al, 2007	- university students, Edinburgh	students: n=2006	- Sexual activity increased the risk of type 2 infection, but the increase in risk with number of sex partners was less consistent	- risk factors for infections with EBV genotypes 1 and 2 are probably different
Correia et al, 2017	- healthy young adults (mainly Caucasians), Britain	n=254 persons (53 positive for EBV DNA in the saliva)	- genotype 2 detected in 2 persons of caucasian background<br>- genotype 1 and 2 coinfections detected in 4/53 persons, all of Asian descent	- genotype 2 is present in a<br>small percentage of Caucasian healthy young adults from Britain

Banko et al, 2012	- heterogeneous patient group, Serbia	IM: n=30<br>Transplanted persons: n=6<br>NPC: n=16<br>HL: n=1	- both genotypes 1 and 2 were detected<br>- genotype 1 was detected in 90.7% samples	- predominance of EBV genotype 1 in diverse Serbian population
Banko et al, 2016	- IM patients, Serbia	IM patients: n=128 (33 EBV DNA-positive serum)	- genotype 1 was present in 96.87% samples (31/32) and genotype 2 in 3.13% samples (1/32).	- predominance of EBV genotype 1 in IM patients from Serbia
Wang et al, 2021	- Lymphoma patients, Inner Mongolia Autonomous Region, China	non-HL: n=46<br>HL: n=16	- EBV genotype EBV was detected in 98.6% of patients (68/69) with genotypes 1 and 2 coinfection detected only in one patient	- predominance of EBV genotype 2 in lymphoma patients from China
Palma et al, 2013	- HL, Mexico	Children (n=42) and adults (n=16) with HL (lymph node biopsy)	- high frequency of type 2 infection (47.6% in children and 69.2% in adults) compared with type 1 infections (33.3% in children and 30.8% of adults) with mixed infections present in 19% of children	- frequent detection of EBV genotype 2 in both adult and pediatric HL patients from Mexico with coinfections detected in pediatric population
Salahuddin et al, 2018	- lymphoma patients, Pakistan	lymphoma patients: n=73 (lymph node biopsy)	- EBV genotype 1 was found in 90.7% of samples, genotype 2 was detected in 9.3% of patients	- predominance of EBV genotype 1 in lymphoma patients in Pakistan
Kafita et al, 2018	- lymphoma patients, Zambia	- lymphoma patients: n=150 (80% NHL, 20% HL)	- EBV DNA was present in 51.8% of samples, genotype 1 was present in all samples except in one with genotypes 1 and 2 coinfection	- predominance of EBV genotype 1 in patients with nHL and HL from Zambia
Tabibzadeh et al, 2021	- patients with malignant diseases (EBV-associated), Iran	- patients (malignant diseases): n=34 (8 with HL), peripheral blood	- EBV genotype 1 was detected in 91.2% of samples	- predominance of EBV genotype 1 in lymphoma patients in Pakistan

eBL - Endemic Burkitt's lymphoma, MSM - men who have sex with men, IM - infectious mononucleosis, NPC - nasopharyngeal carcinoma, HL - Hodgkin's lymphoma, NHL - non-Hodgkin's lymphoma, HIV-1 - human immunodeficiency virus type 1.

Table S4. Distribution of EBV LMP1 variants in patients with EBV-associated diseases.

Study	Experimental Model	Study Design	Main Results	Conclusions
Knecht et al, 1993	HL, persons of European descent (20 Swiss, 16 French, 14 Danish), 2 persons from Libya	HL: n=52	- wild type was present in almost all samples (90.4%)<br>- LMP1 30 bp deletion variant (similar or identical to COA and 1510 isolates detected in Asian NPC patients) was associated with a high frequency of HRS (including anaplastic cells) and necrotic areas	- LMP1 30 bp deletion variants are present in a small proportion of HL patients of European descent and are associated with severe histopathology
Sandvej et al, 1994	HL, PTL, IM from Denmark and Malaysia	HL: n=62; PTL: n=19; IM: n=9 (tonsils) from Denmark	- LMP1 30 bp deletion variant was detected in 16/56 cases of HL (28%), 11/18 (61%) cases of Danish PTLs, all cases of Malaysian PTLs, and 3/9 (33%) cases of IM	- LMP1 30 bp deletion variants are frequent in HD, PTL and IM patients of European descent and in all PTLs of Asian patients

		PTL (Malaysia): n=13 (paraffin blocks)		
Chang et al, 1995	TL, NPC, HL, BCL, healthy persons from Taiwan,	TCL: n=7<br>HL: n=2<br>BCL: n=1<br>Healthy patients: 4 lymph nodes, 40 samples of nasopharyngeal tissue, 78 throat washings	- LMP1 30 bp deletion variant and loss of an XhoI site was detected in 56/57 tumor-tissue samples, in 92% of EBV-positive normal nasopharynx tissues and 84% of EBV-positive throat washings of healthy individuals	- exceptionally high frequency of LMP variant with 30 bp deletion and loss of XhoI site in patients with malignant diseases and healthy persons from Taiwan
Khanim et al, 1996	LCLs from healthy EBV-seropositive persons and 4 from IM patients	LCL: n= 92 (22 of European origin, 39 of African origin, 11 from the New Guinea, 10 from China) and n=4 (IM)<br>- IM: n=8 (lymph nodes and palatine tonsils)<br>- HL: n=25 (UK)<br>- NPC: n=10 (Hong Kong), n=11 (Caucasian, UK)<br>- GC: n=4 (UK)	- LMP1 30 bp deletion variant was detected in all non-malignant patients of European descent and in 13/30 (40%) of patients with malignant diseases and in 9/22 (41%) LCLs<br>- *Xho*I polymorphism was not detected in European IM, HD, NCP and GC patients, and was present in only 2 LCLs<br>- high frequency of LMP1 variant carring both *Xho*I polymorphism and the LMP1 30 pb deletion in Asian (Chinese) population, both with NCP and healthy persons	- LMP1 variant carring *Xho*I polymorphism and the LMP1 30 pb deletion predominates in Asian population<br>in Asian (Chinese) population<br>- LMP1 30 bp deletion variant in all patients of European and African origin does not contain *Xho*I polymorphism
Dirnhofer et al, 1999	cHD and reactive lymphoid tissue, Mexico	- cHL (n = 57) and reactive lymphoid tissues (n = 20)	- LMP1 30 bp deletion variant was found in 28/35 (80%) EBV-positive HD, 9/9 (100%) HD with H-RS cells negative for EBV and in 10/17 (59%) reactive lymph nodes with overall prevalence of 73%	- the study reporting the highest incidence of LMP1 30 bp deletion variant in HL
Banko et al, 2012	- heterogeneous patient group, Serbia	IM: n=30<br>Transplanted persons: n=6<br>NPC: n=16<br>HL: n=1	- EBV LMP1 variant distribution in the samples was: B95-8 (wild type) (32.1%), China 1 (24.5%), North Carolina (NC; 18.9%), and Mediterranean (Med; 24.5%)<br>- the presence of LMP1 variants with 30 bp, rare 69 bp, or previously uncharacterised 27 bp deletions were not related to malignant or non-malignant isolate origin	- diverse LMP1 variant distribution in patients with malignant and non-malignant diseases
Correia et al, 2017	- healthy young adults (mainly Caucasians), Britain	- healthy persons: n=254 (53 positive for EBV DNA in the saliva)	- B95-8, China 1 and 2, Mediterranean and North Carolina EBV LMP1 variants were detected in the samples	- a heterogeneous pattern of LMP1 variant distribution in healthy young adults from Britain
Alves et al, 2022	- healthy persons and patients with	- healthy persons: n=42	- LMP1 variants detected included Mediterranean (40.2%, n = 33),	- a specific pattern of LMP1 variants with a

	malignant diseases, Brasil	- malignant diseases: n=33 (cHL: n= 26; BL: n=7; reactive hyperplasia n=4)	Raji/Argentine (39%, n = 32), B95-8 (6.1%, n = 5), and Asian II (1.2%, n = 1)<br>- Raji/Argentine clade was predominantly clustered with lymphomas (61%) and the Mediterranean clade with non-malignant cases (59%) (p = 0.1)<br>- the Raji/Argentine clade carring with polymorphisms I124V/I152L, del30 bp, and ins15 bp was present in 61% of lymphomas	characteristic pattern of variants in lymphoma
Montes-Mojarro et al, 2020 (review)	ENKTL, Mexico, China, Peru, Argentina (Elenitoba-Johnson et al, 1998; Chiang et al, 1999; Thorley-Lawson et al, 2015; Montes-Mojarro et al, 2021)	- ENKTL: n=140	- a predominance of the B95-8 wild type (52.1%) with a 37.1% of patients harboring 30 bp deletion	- mutational landscape of ENKTL in Latin America includes LMP1 30 bp deletion variants

Hl - Hodgkin's lymphoma, HRS - Hodgkin and Reed-Sternberg cells, PTL - peripheral T-cell lymphoma, IM - infectious mononucleosis, TCL - T-cell lymphoma, NPC - nasopharyngeal carcinoma, BCL - B-cell lymphoma, LCL - Lymphoblastoid cell line, BL - Burkitt's lymphoma.
